# Plant Coilin: Structural Characteristics and RNA-Binding Properties

**DOI:** 10.1371/journal.pone.0053571

**Published:** 2013-01-08

**Authors:** Valentine Makarov, Daria Rakitina, Anna Protopopova, Igor Yaminsky, Alexander Arutiunian, Andrew J. Love, Michael Taliansky, Natalia Kalinina

**Affiliations:** 1 A. N. Belozersky Institute of Physico-Chemical Biology, Moscow State University, Moscow, Russia; 2 Biological Faculty, Moscow State University, Moscow, Russia; 3 Physical Faculty of Moscow State University, Moscow, Russia; 4 The James Hutton Institute, Invergowrie, Dundee, United Kingdom; Weizmann Institute of Science, Israel

## Abstract

Cajal bodies (CBs) are dynamic subnuclear compartments involved in the biogenesis of ribonucleoproteins. Coilin is a major structural scaffolding protein necessary for CB formation, composition and activity. The predicted secondary structure of *Arabidopsis thaliana* coilin (Atcoilin) suggests that the protein is composed of three main domains. Analysis of the physical properties of deletion mutants indicates that Atcoilin might consist of an N-terminal globular domain, a central highly disordered domain and a C-terminal domain containing a presumable Tudor-like structure adjacent to a disordered C terminus. Despite the low homology in amino acid sequences, a similar type of domain organization is likely shared by human and animal coilin proteins and coilin-like proteins of various plant species. Atcoilin is able to bind RNA effectively and in a non-specific manner. This activity is provided by three RNA-binding sites: two sets of basic amino acids in the N-terminal domain and one set in the central domain. Interaction with RNA induces the multimerization of the Atcoilin molecule, a consequence of the structural alterations in the N-terminal domain. The interaction with RNA and subsequent multimerization may facilitate coilin’s function as a scaffolding protein. A model of the N-terminal domain is also proposed.

## Introduction

Cajal bodies (CBs), subnuclear compartments physically and functionally associated with nucleoli, participate in the maturation of splicing small nuclear ribonucleoproteins (snRNPs) and facilitates their modification and assembly *via* small CB specific RNAs (scaRNAs). In addition, CBs associate with snRNA genes, histone gene clusters, Gems, and PML bodies, and also participates in the biogenesis and delivery of telomerase to telomeres. CBs are most frequently detected in cells with high transcriptional demands, such as neuronal and cancer cells or in cell lines infected by viruses (see review by [1]).

Coilin, a major structural scaffolding protein necessary for CB formation, composition and activity, is predominantly found in CBs, but is also distributed throughout the nucleoplasm [2], [3], [4]. Coilin orthologues have been identified by sequence comparison and cloning in *Xenopus* [5], mouse [6], *Arabidopsis* [7] and *Drosophila* [8] and have been shown to localize in prominent nuclear bodies in these organisms. Although coilin is not strongly evolutionarily conserved with respect to amino acid (aa) sequence or size, mammalian (human and mouse) coilin proteins are the exception as they have similar molecular masses (near 60 kDa) and a high homology ratio of about 67% overall and more for the conserved regions [6], [9]. In contrast, the homology between human and *Xenopus* coilin is only 42%, with human coilin having 576 amino acid residues compared with the 508 residues found in *Xenopus* coilin [6], [10]. In invertebrate species the level of homology is poor, such that it has restricted the identification of new coilin genes in these organisms. This may explain why coilin genes have not been found in some widely utilized model organisms such as *Caenorhabditis* and *Saccharomyces* [11].

Recent knockout and knockdown studies have demonstrated that coilin is necessary for proper CB formation, composition and activity [8], [12], [13], [14]. Coilin knockdown in HeLa cells has been shown to reduce cellular proliferation [15], [16], presumably due to depleted snRNP resources. Coilin gene knockouts in mice produce a sublethal phenotype, typified by either mortality in a proportion of the homozygotes or induction of serious reproductive problems in those that survive [13]. In contrast, homozygous Ncb-1 *Arabidopsis thaliana* mutants that have lesions in the coilin gene are completely viable even though they cannot form CBs [14]. Similar results were observed in coilin-deficient mutants of *Drosophila*, where the absence of CBs has no effect on their survivability [8].

Two important regions with sequence conservation have been identified in coilin proteins from different species [6]. These regions encompass ∼100 aa residues at both the N- or C-terminal part of the protein, the positions of which may vary depending on the species from which the coilin is derived. The highly conserved N-terminal domain [17] facilitates coilin self-interaction and oligomerization [18]. Recent work has demonstrated that the C-terminal conserved region contains a Tudor-like domain, which in the case of human coilin is situated between 460 and 560 aa residues [19]. This domain is atypical in that it bears two large unstructured loops. The C-terminal part of human coilin also contains a region rich in arginines and glycines (the RG box or repeat), which is N-terminal to the Tudor-like domain [19], [20], [21]. In the central part of the protein between the N- and C- terminal domains, two nuclear localization signal sequences (NLS1 and NLS2) and a presumable nucleolar localization signal (NoLS) have been identified [18]. Interestingly, the final 10 residues of the most distal end of the C-terminus are implicated in controlling availability of the N-terminus for self-interaction, influencing CB formation and number [17], and modulating coilin localization [22]. Unfortunately to date, the domain organization and structural aspects of coilin remain unelucidated.

Coilin interacts directly with the survivor motor neuron (SMN), U snRNPs and Sm proteins [23]. The binding of SMN is mediated by the RG box [20], whereby symmetrical dimethylation of the arginine residues increases SMN/coilin interaction [24]. Interestingly, *Arabidopsis thaliana* coilin (Atcoilin) does not contain an RG box, and it is currently not known whether any of the arginine residues in Atcoilin are dimethylated. Similarly, no homologue of SMN has currently been identified in the *Arabidopsis* genome [7]. Sm protein and U snRNP binding requires the C-terminal 156 residues of human coilin [23]. Coilin is a constitutive phosphoprotein that is hyperphosphorylated during mitosis [25]. Phosphorylation of coilin also appears to impact its ability to interact with SMN and Sm proteins: SMN preferentially binds to hypophosphorylated coilin but SmB’ binds more to phosphorylated coilin [21]. Additionally, coilin interacts with Ku proteins and can inhibit in vitro non-homologous DNA end joining [26], suggesting that nucleoplasmic coilin may have a role in stress response pathways such as those caused by DNA damage. There is not much data concerning the characteristics of coilin interaction with nucleic acids, though the N-terminal part of human and *Xenopus* coilins was shown to bind ssDNA and poly r(G) *in vitro* [3]. Recent data demonstrated that human coilins co-purified with RNA and DNA, interacted with dsDNA *in vitro* and might take part in snRNA processing [27].

In this work we demonstrate that coilin proteins from different origins share a similar structural organization, which enabled us to reveal three structural domains within the *Arabidopsis thaliana* coilin molecule. The isolated domains were expressed as recombinant proteins and the physical and RNA-binding characteristics were elucidated, allowing identification of the sites responsible for the RNA-binding activity.

## Materials and Methods

### CD Spectroscopy

Protein samples at a concentration of 100 µg/ml in 1 mM phosphate buffer pH 7.5 were loaded into 1–2 mm cells, and CD spectra were recorded from 185 to 250 nm at 25°C in a “Chiroscan” CD spectrometer (“Applied Photophysics”, England). The CD spectra were recorded at a speed of 0.5–1.0 nm/s with base-line subtraction. The measured spectra were smoothed using the instrument software. [θ] value calculations were based on the mean amino acid residue molecular weight of 110.

### Fluorescence Spectra

Coilin proteins (0.03 mg/ml) or protein-RNA complexes in 1 mM phosphate buffer pH 7.5, were loaded into 1 cm cells of a FluoroMax (HORIBA Jobin Yvon, USA) spectrofluorimeter. Samples were excited at 280-nm and emission spectra were recorded in the 300- to 400-nm range. Readings took place at 25°C.

### Dynamic Light Scattering

Protein samples at 0.05 mg/ml in 1 mM phosphate buffer pH 7.5 were loaded into 1 cm cells of the Zetasizer Nano ZS (Malvern Instruments, UK) dynamic light scattering device, and measurements were obtained using the He-Ne laser (633 nm). Curves were fitted using Dispersion Technology Software (DTS) version 5.10.

### Cloning and Mutagenesis

The mutant variants of coilin protein were constructed using the *Arabidopsis thaliana* sequence (NM_101173.4; GI:42562030). The deletion mutants were produced via PCR amplification of the desired sequence and cloned into the PQE30 expression vector using SphI and SalI restriction sites. The resulting proteins contained a 6×histidine sequence on the N-terminus. The substitution mutations (replacement of R or K with Alanines) were created using overlap PCR.

### Expression and Purification of the Proteins

The *E. coli* expression strain *JM109* was transformed with the plasmids, and the proteins of interest were expressed by adding 1 mM IPTG to a 1/20 diluted overnight culture, which was further shaken at 37°C for 2 h. Proteins were purified under denaturing conditions using Ni-NTA Qiagen agarose (Qiagen, West Sussex, UK), according to the manufacturer’s protocol.

For structural studies and RNA-binding assays the recombinant proteins were renatured and the urea was removed by dialyzing the 0.1 ml sample in MilliQ water for 1.5 h at room temperature with 5 changes of water. Samples were spun to clear by centrifugation for 10 min at 10 000 rpm.

### RNA Binding Assay

RNA-binding activity of the proteins was tested using a gel-shift approach. RNA and protein were mixed at various ratios in RNA binding buffer (20 mM Tris-HCl pH 7.5; 1 mM DTT; 3 mM MgCl_2_; 50 mM NaCl), and after incubation on ice for 15 min samples were loaded onto Tris-acetate agarose gels stained with ethidium bromide or Sybr Gold Nucleic Acid Gel Stain (Invitrogen). Gels were photographed and the amount of free non-retarded RNA was quantified using Gel-Pro Analyzer (Version 3.1.00.00, Media Cybernetics). U1 snRNA, U2 snRNA and pGEM7 RNA substrates were prepared by T7 transcription of PCR products containing a T7 promoter adjacent to the corresponding DNA sequence.

### Atomic Force Microscopy

The protein solution was allowed to adsorb onto the surface of freshly cleaved mica for 1 minute, after which the solution was then carefully removed with filter paper. This substrate was immediately placed on to a drop of double-distilled Millipore water (this procedure was repeated twice), and the surface was then air dried. This sample preparation method was used to eliminate any remaining salts and minimize artifactual aggregation during drying. Atomic force microscopy (AFM) analysis was performed on these samples using a Multimode AFM with a Nanoscope IIIA Controller (Digital Instruments, USA) in tapping mode with a typical scan rate of 1 Hz. The measurements were performed in air in tapping mode using sharp silicon cantilevers (NT-MDT, Russia) with a guaranteed tip radius of 10 nm.

## Results

### The Bioinformatic Analysis of the Atcoilin Amino Acid Sequence

**Figure 1:**
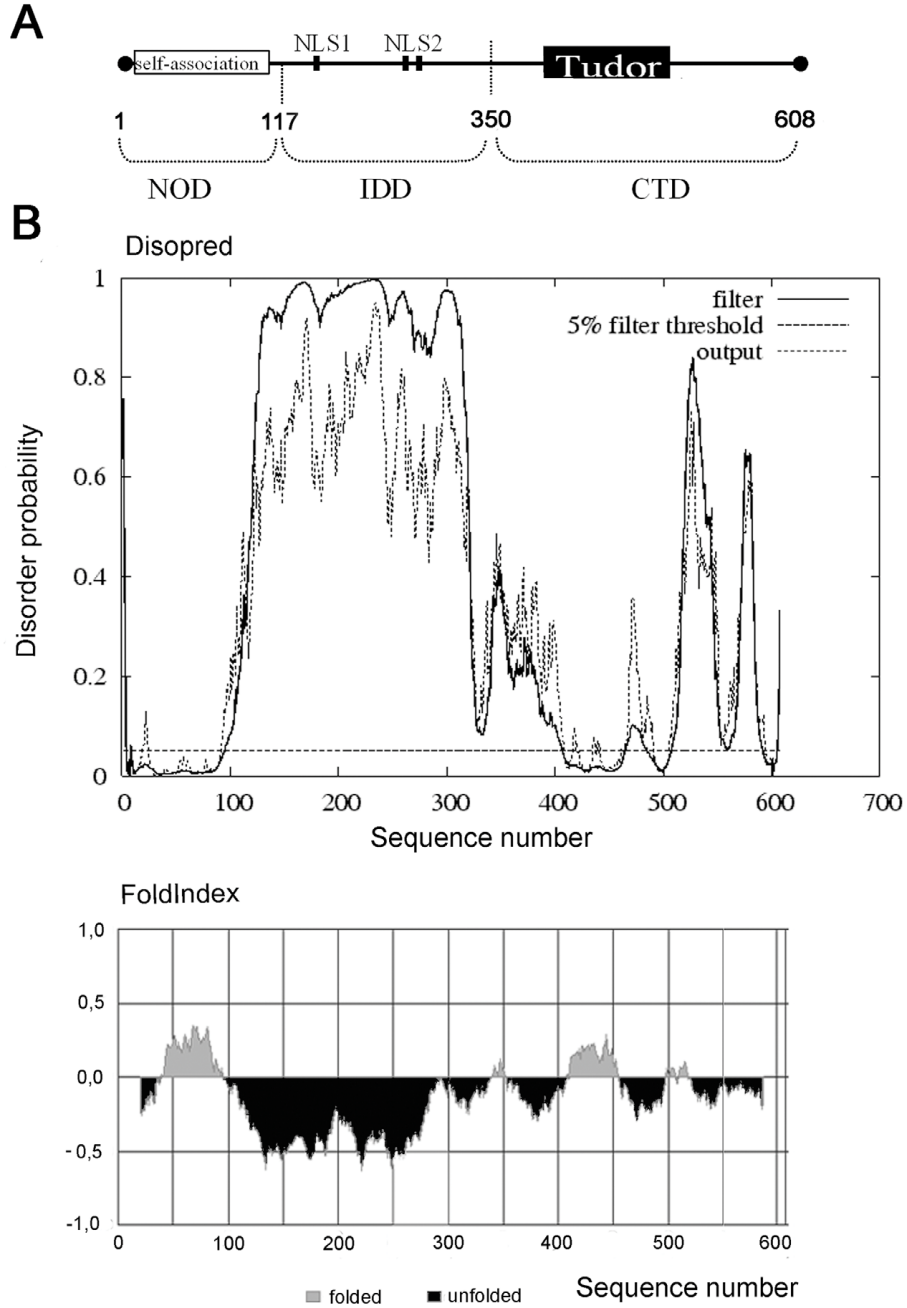
Amino acid sequence analysis and predicted domain organization of *Arabidopsis thaliana* **coilin.** (**A**) Schematic representation of functional sites and regions that have been identified within the coilin protein molecule. (**B**) The predicted domain organization of *Arabidopsis thaliana* coilin, as elucidated by the bioinformatic tools FoldIndex and DISOPRED.

The schematic representation of coilin from *Arabidopsis thaliana* (NM_101173.4; GI:42562030), Atcoilin, a 608-amino acid protein [7] which shares functional and structural characteristics with vertebrate (human) coilin, is shown in Figure 1A. As demonstrated by [7], there are two regions with high homology between the vertebrate and plant coilins: the N-terminal 100 amino acid residues, and the C-terminal 100 amino acid region encompassing a Tudor-like domain (corresponding to the C-terminal residues 460–560 and 410–510 of human coilin and Atcoilin respectively). The N-terminal region is suggested to be responsible for the self-association of the protein [18]. Similar to human coilin, two NLSs are predicted in the central part of the protein [7].

There are several indications which may point to the presence of intrinsically disordered regions within coilin molecules. Firstly, it has been reported that coilin molecules from different organisms have unexpected electrophoretic mobilities, whereby the *M*r and the predicted MW do not correlate [6], [8]; a phenomenon observed with human, mouse and *Xenopus* coilin proteins. This is also true of Atcoilin which has a *Mr* near 90 kDa, although it has a predicted MW of 68.7 kDa. Secondly, there have been several unsuccessful attempts to produce stable full-length human coilin crystals [19]. In order to elucidate the level of order/disorder, the amino acid sequence of Atcoilin was analyzed using the FoldIndex and DisoPred web-services [28], [29]. Figure 1B shows that the central part of the protein is predicted as being highly disordered, with additional unfolded regions identified in the C-terminus. In contrast, the conservative N-terminal region, and parts in the C-terminal portion thought to correspond to a Tudor-like domain structure were predicted to be folded (Figure 1B).

Using bioinformatic predictions of coilin structure, we suggest that Atcoilin contains two structural domains in the N-terminal part including an ordered domain on the N terminus (NOD) and a central internal disordered domain (IDD) and at least one domain in the C-terminal part (C-terminal domain, CTD) with a presumable Tudor-like structure abutting an intrinsically disordered C-terminus (Figure 1A).

Interestingly, despite the significant difference in the amino acid sequences, a similar pattern of ordered/disordered regions was identified in coilin proteins from different organisms. For example, coilin proteins from humans, *Danio rerio* and *Xenopus laevis* share a structured N-terminal region (about 100 aa), a long central disordered region and a C-terminal region which contains ordered and disordered parts (Figure S1).

### The Structural Properties of Atcoilin and its Presumable Domains Revealed by Circular Dichroism and Tryptophan Fluorescence Methods

**Figure 2:**
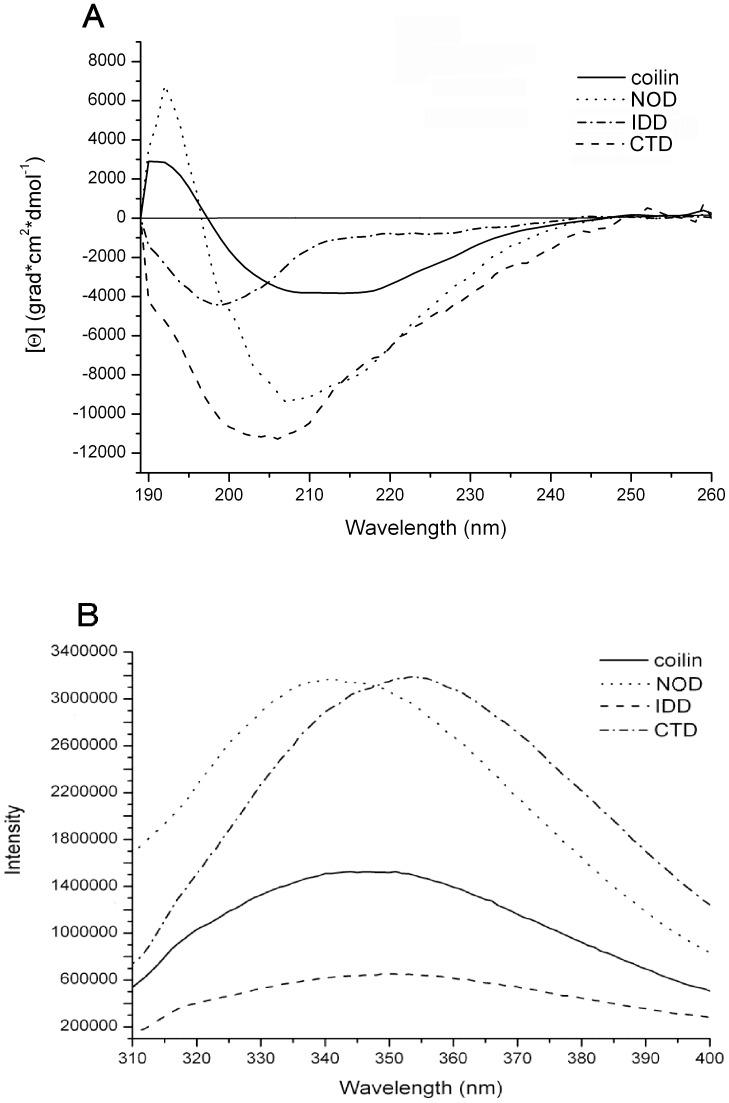
Structural characteristics of the predicted domains of Atcoilin. (**A**) Circular dichroism far UV-light spectra of the wt Atcoilin and its isolated domains. (**B**) Tryptophan fluorescence spectra of the wt Atcoilin and its isolated domains. Fluorescence intensity is given in relative units.

The recombinant proteins corresponding to wild type (wt) Atcoilin and its presumable NOD (1–117 aa residues), IDD (117–350 aa residues) and CTD (350–608 aa residues) domains (Figure 1A) were expressed and purified from *E. coli* (Figure S2). Their secondary structure was analyzed using a circular dichroism (CD) approach (Figure 2A).

The CD spectrum of wt coilin has two negative maxima at 208 nm and 217 nm and also has a weak signal intensity; a curve trend typical of proteins that contain a considerable amount of β-structural elements [30]. The quantification of the spectrum by the K2D2 web-service [31] indicated that the wt coilin is composed of 30% β-strands and 16% α-helices (Table 1). It is necessary to mention that the determination of the content of β-structure elements (in contrast to α-elements) by algorhithms calculating the data achieved from circular dichroism is not very precise and should be validated by the form of spectre curve and overall signal intensity [30], [32], [33]. The CD spectrum of NOD has a negative peak at 208 nm and a positive peak at 190 nm, suggesting significant α-helical element content (about 30%), but lower quantities of β-strand structures (13%) within this domain (Table 1) [30]. The IDD spectrum is quite different: the single negative maximum at 200 nm indicates that this domain contains long completely unfolded regions, and moreover, the overall low signal intensity suggests that β-strands are present in insignificant amounts judging from the form of the curve [32], [33]. According to the K2D2 quantification, CTD has low α–helical content but contains β-strands to around 22%, which is in good agreement with the amount of secondary structure elements within the Tudor-like domain of human coilin (Table 1) [19]. The shift of a negative peak from 208 nm (which is characteristic for α-proteins) to 200 nm demonstrates that alongside the ordered secondary structure elements, the C-terminal domain also contains substantial unfolded regions, which likely correspond to the big loops between elements of the Tudor-like domain and also the long unfolded C-terminus.

**Table 1:** Content of secondary structure elements in the coilin molecule*.

Protein	α %	β %	Disordered %
Coilin	16	30	54
NOD	30	13	57
IDD	7	29	64
CTD	8	22	70

* - Determination of the content of β-structure elements (in contrast to α-elements) by algorhithms calculating the data achieved from circular dichroism is not very precise and can fall far from true. Besides, the inaccurate increase of β-elements drives to inaccurate decrease of non-structured elements within the protein. That is why the calculated amount of β-elements should be validated by the form of spectre curve and overall signal intensity [30], [32], [33].

A tryptophan fluorescence approach was used to determine the packing density of the Atcoilin molecule (Figure 2B). This method allows evaluation of the local environment of tryptophan residues (hydrophilic or hydrophobic) and elucidates the lability of tryptophan residues in the protein molecule [34], [35]. The spectrum of tryptophan fluorescence of the wt coilin has a peak at 347 nm, indicating both the hydrophilic environment of the tryptophan side chains and the poor formation of a globular hydrophobic core in the protein molecule. The central and C-terminal domains of coilin have fluorescence maxima at 350 nm for IDD and 354 nm for CTD, which indicates an even greater level of hydrophilicity in the local tryptophan environment. In contrast, with NOD the fluorescence peak is observed at 342 nm, suggesting a more hydrophobic environment of the tryptophan residues in comparison with the other two domains and wt protein.

In addition to the wavelengths at which maxima occur, it is necessary to take into account the comparative intensity of the fluorescence signal. NOD and CTD provide the peaks of pretty close intensity, despite the fact that the NOD has only one tryptophan residue compared to 6 tryptophans found in CTD. With CTD, the high signal despite the hydrophilic position of the maximum may be explained by the non-involvement of tryptophan residues in the formation of its tertiary structure. All these residues are localized in unfolded regions of the CTD: one is within a loop between the Tudor fold β-sheets, two are more upstream, and the remainder is in the disordered extreme C-terminus. The intensity of the fluorescence signal of wt coilin is much lower, whereas that of central IDD is the lowest. These data might indicate that NOD has the most stable structure, while IDD and CTD domains are rather labile with significant amounts of disorder. The intensity and maximum of the wt coilin fluorescence differs from that of the isolated domains. The fluorescence peak position of the wt coilin is nearly intermediate between the peak maximums of NOD and CTD (Table 2). Similarly, the intensity of the wt coilin fluorescence is more than that of IDD, but less than that of NOD and CTD, demonstrating that the spectrum of the wt coilin might represent combined spectra of all three domains. Comparison of these characteristics points to the fact that the structure of all three isolated domains is likely the same as their structure within the full-length wt protein molecule.

**Table 2:** Influence of U1 snRNA on coilin structure.

Protein	λmax without RNA	Λmax with RNA
Coilin	347	340
NOD	342	336
IDD	350	350

Thus, the tryptophan fluorescence and CD data verifies the various degrees of disorder in the different coilin domains and supports the suggestion that coilin is a multidomain protein with a high degree of intrinsic disorder.

### RNA-binding Activity of Coilin and the Sites Providing this Activity

Coilin is a nucleic acid-binding protein, as shown by its interaction with ds/ss DNA, poly r(U) and poly r(G) [3], [27]. The coilin C-terminal domain binds directly to protein moieties of U2, U4, U5 and U6 snRNPs, but not of U1 and U7 snRNPs [23]. To the best of our knowledge the direct interaction between coilin and snRNAs has never been studied.

**Figure 3:**
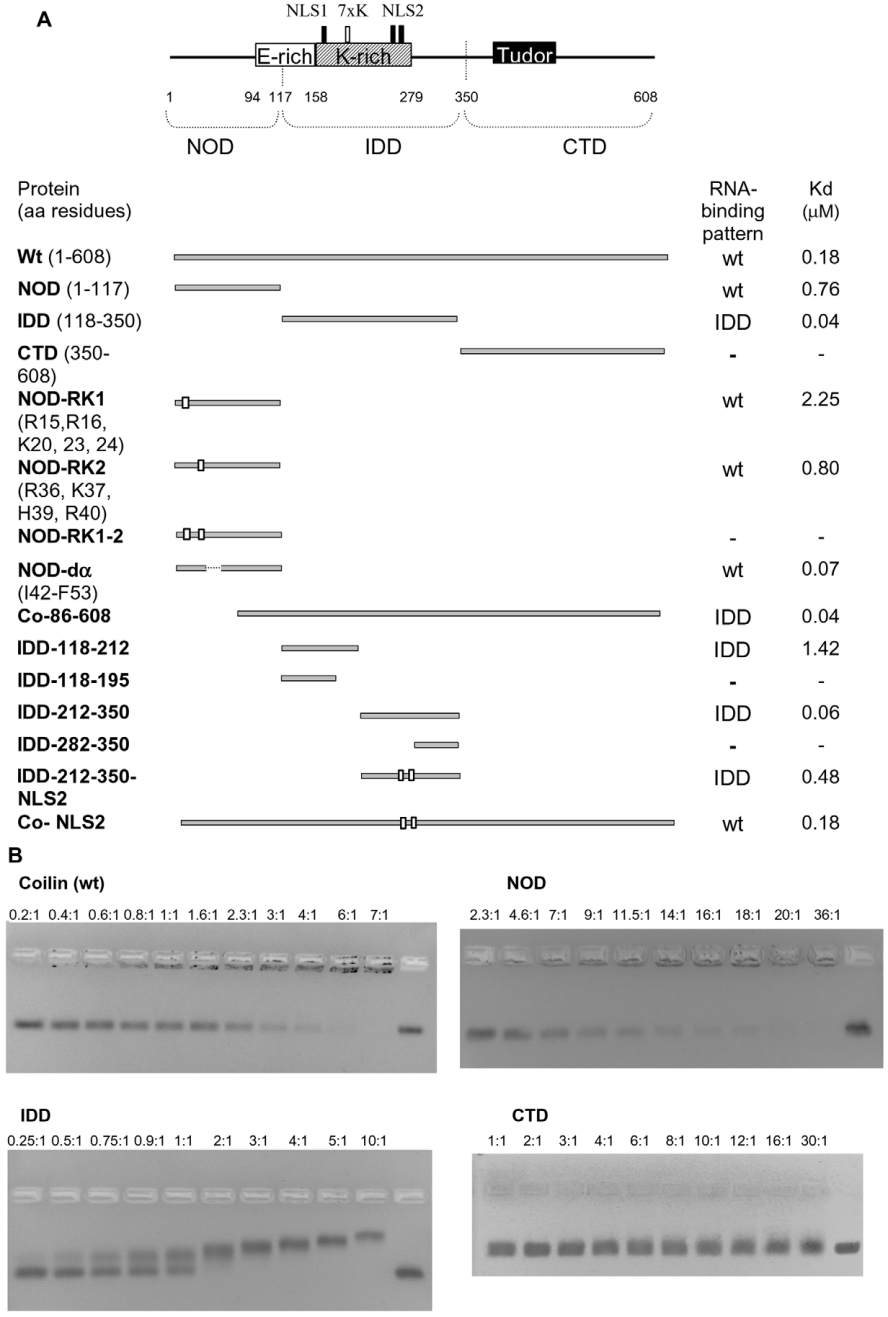
Atcoilin mutants and their RNA binding capacities. (**A**) Schematic representation of Atcoilin and its mutants. The indicated protein regions and motifs are according to MyHits Motif Search, and the domain predictions are according to FoldIndex and DisoPred. The substitution mutations (R and K to A) are indicated by white boxes and the RNA-binding pattern and apparent *Kd* value for each mutant is indicated. (**B**) RNA binding capacity of Atcoilin and its isolated domains, as determined using EMSA. Increasing amounts of protein (protein:RNA ratios indicated above each lane) were incubated with 0.1 µg of RNA in RNA binding buffer (see Materials and Methods) and loaded onto 2% non-denaturing Tris-acetate agarose gels. The rightmost lane contains RNA without protein.

In this report we have tested the ability of coilin to directly interact with two snRNAs - U2 snRNA and U1 snRNA and a non-specific artificial RNA (160 nt). In the electrophoretic mobility shift assay (EMSA) the recombinant Atcoilin is able to bind all these substrates with similar characteristics: apparent *Kd* values are 0.18–0.2 µM, with 50% of the RNA being incorporated into the complex at the protein:RNA molar ratio of 2∶1, and 100% of the RNA was bound at the molar ratio of 6∶1 (Figure 3B, 4A). The Hill constant is about 1.2±0.1, and is indicative of low cooperative binding.

**Figure 4:**
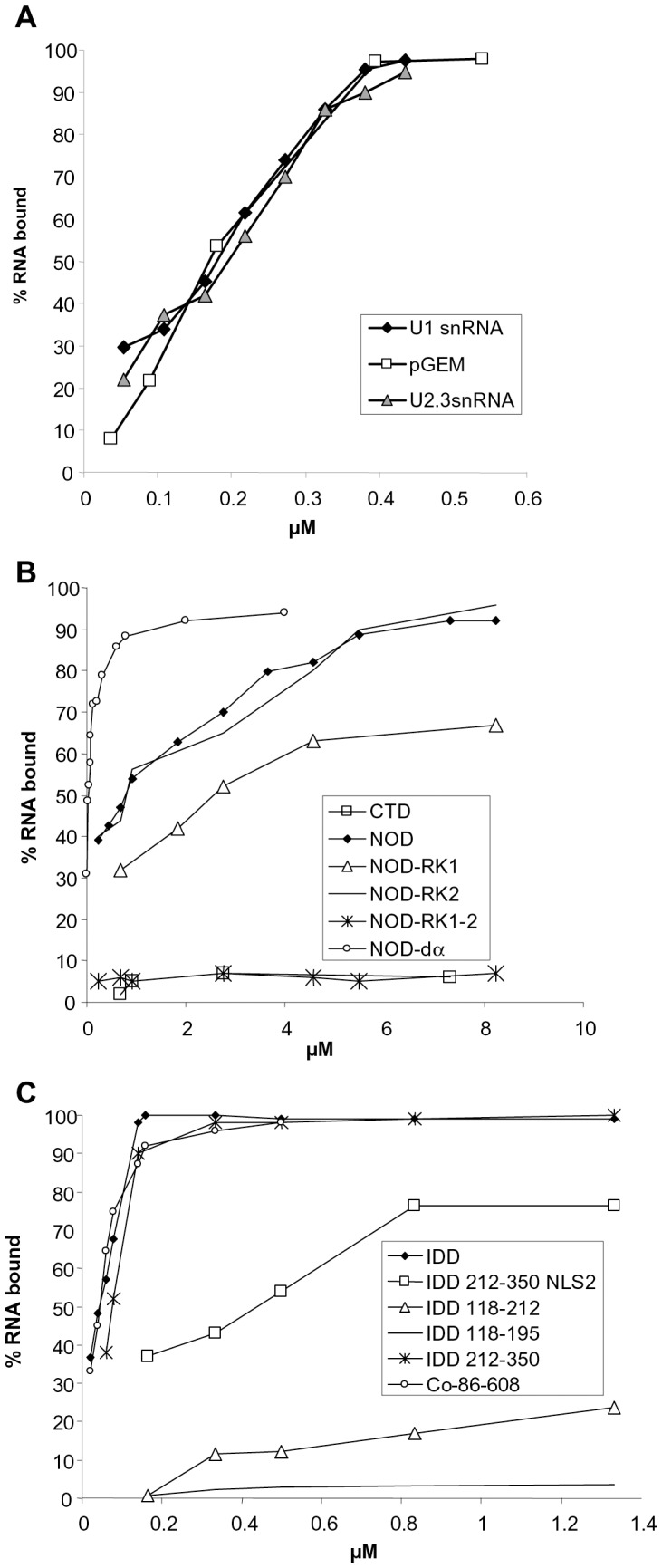
Analysis of the RNA-binding activity of Atcoilin and its mutants. The percentage of RNA complexed with the protein is plotted versus the protein concentration in the sample (µM). (**A**) Coilin binding to U1 and U2 snRNAs and pGEM7 RNA. (**B**) and (**C**) Binding of U1 snRNA by NOD, CTD, IDD and their mutants. Mutants compared on one plot are indicated.

To identify the regions responsible for RNA interaction, the isolated domains of Atcoilin have been tested for their ability to bind U1 snRNA. CTD demonstrates no RNA-binding activity (Figure 3B, 4B), whereas the other two domains effectively bind RNA, albeit in different manners. The NOD pattern of RNA binding is similar to that of the full-length Atcoilin; RNP complex migration is retarded in the wells of the agarose gel. However, for NOD, the apparent *Kd* value is about 0.76 µM and full RNA binding is achieved at the high protein:RNA molar ratio of 36∶1. The higher *Kd* value of NOD could be due to stereochemical obstacles; in the high-order oligomer that this domain forms some of the RNA-binding sites may be deeply embedded in the complex and consequently they may not be able to interact with RNA. In contrast, the IDD complexes with RNA enter into the agarose gel. In this case no free RNA remains in the gel at the protein:RNA molar ratio 2∶1. Further addition of the protein gradually increases retardation of the complex. The apparent *Kd* (0.04 µM) is lower than that of the wt protein (Figure 3A). These results suggest that coilin has at least two RNA-binding sites.

To characterize these sites we mutated two RNA-binding domains of Atcoilin. The deletion of the C-terminal 47–117 aa of NOD has no effect on RNA-binding activity, while the deletion of the N-terminal 1–45 aa abolishes it completely (data not shown). Since the RNA-binding of NOD is provided by its N-terminal part, we removed some basic amino acids within it. The deletion of the N-terminal highly conservative (between humans and *Arabidopsis*) sequence (aa 1–13) including arginine residues R7 and R9 (Figure S3A) has no effect on RNA binding. In contrast, the substitution of several positively charged residues (R15, R16, K20, K23, K24) with alanines (mutant NOD-RK1) results in decreased RNA binding (*Kd* was about 2.25 µM –3 times more than that of NOD) (Figure 3A, 4B). The sequence containing these residues was not found in vertebrate coilin homologs (Figure S3A).

Alignment of Atcoilin to the human coilin reveals a common pattern they share: 34RCR36 in human coilin, which corresponds to the 36RKCHR40 sequence in Atcoilin (Figure S3A). Replacement of the positive amino acids in this pentamer with alanines does not have any pronounced effect on RNA binding by NOD (mutant NOD-RK2, Figure 3A, 4B). However, the combination of two replacements (RK1 and RK2) abolishes the NOD RNA-binding activity (mutant NOD-RK1-2, Figure 3A, 4B), demonstrating that these two sets of positively charged amino acids provide the RNA-binding activity of NOD.

Within the IDD several sites were identified using MyHits Motif Scan (Figure 3A). The region comprising amino acid residues 158–279 is Lysine-rich and contains two potential NLSs (NLS1 175KRKK178 and NLS2 264KKAKR268). The 202KKKKKKK208 sequence located between them might be a homolog of the human coilin “cryptic NoLS” identified by [18]. Several IDD deletion mutants were constructed to reveal the importance of each of these elements in RNA binding. The IDD-118-195 mutant containing part of the K-region, including the NLS1, showed no detectable binding of U1 snRNA (Figure 3A, 4C). However, weak RNA binding (the apparent *Kd* value of 1.42 uM; Figure 3A, 4C) was detected with the further inclusion of the putative “cryptic NoLS” homolog in this sequence (mutant IDD-118-212). The distal part of IDD (IDD-212-350) provides RNA binding similar to that of the intact IDD with a *Kd* of 0.06 uM (Figure 3A, 4C). Interestingly, the C-terminus of IDD (IDD-282-350) shows no ability to bind RNA (Figure 3A, 4C). Consequently the NLS2 plays a main role as an RNA-binding cluster. Indeed, the substitution of lysines and arginine with alanines in the NLS2 (IDD-212-350-NLS2) severely reduces the RNA-binding activity of the protein, with a *Kd* of 0.48 µM - 12 fold more than that of the intact IDD (Figure 3A, 4C).

To understand the role of the RNA-binding sites we have studied their functionality within the whole coilin molecule. The substitution of NLS2 with alanines (Co-NLS2) produces no change in the RNA binding activity, since the *Kd* is similar to that of the wt protein (Figure 3A). When the N-terminal RNA-binding site is deleted, the resulting mutant (Co-86-608) demonstrates a similar RNA-binding pattern to that of the isolated IDD; the RNP-complexes enter the gel, the protein:RNA molar ratio of full binding is 2∶1 and the *Kd* is about 0.04 µM (Figure 3A). These data suggest that each of the RNA-binding sites can function within the full-length coilin molecule independently. Although the IDD RNA-binding site is likely accessible, the mode of RNA binding characteristic to the full-length coilin is determined by the NOD.

Thus, RNA-binding sites have been identified within the Atcoilin molecule; two positive charged sets in the N-terminal half of the NOD and at least one positively charged cluster (the presumable NLS2) in the IDD. These sites are able to function independently and provide effective RNA binding in a non-cooperative or low cooperative manner.

### The Influence of RNA on Atcoilin Structure and Multimerization

**Figure 5:**
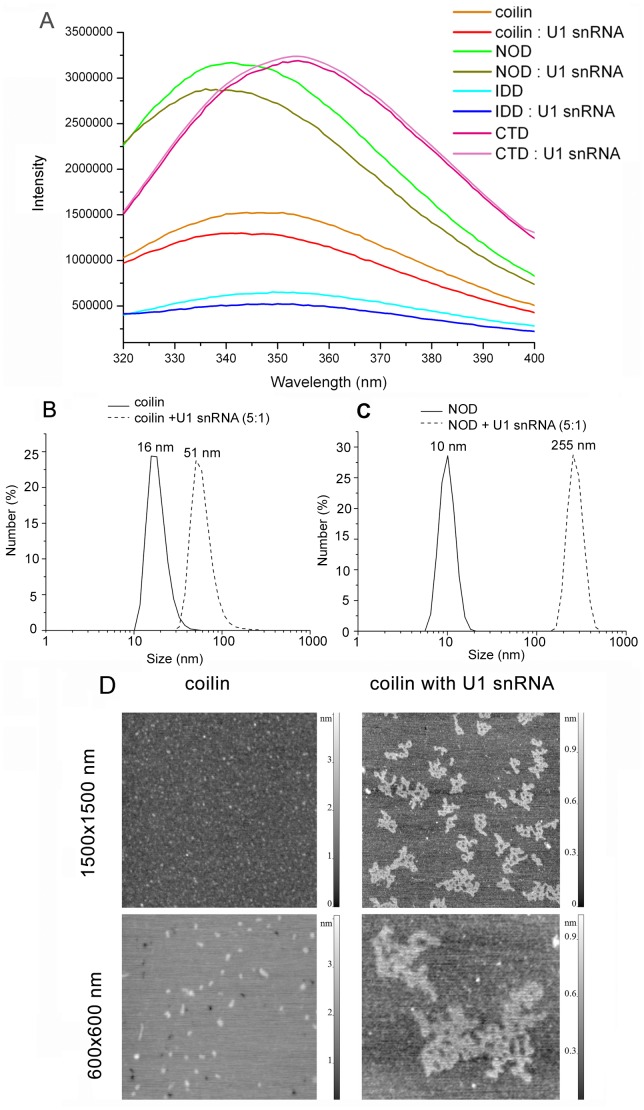
Effect of U1 snRNA on Atcoilin multimerization. (**A**) Effect of U1 snRNA on the structure and packing density of Atcoilin and its RNA-binding domains, determined via tryptophan fluorescence, the intensity of which is given in relative units. (**B**), (**C**), The hydrodynamic radii of (**B**) coilin and (**C**) NOD as free proteins or in complex with U1 snRNA, as elucidated by the DLS method. (**D**) Atomic-force microscopy of coilin as a free protein (left panels) or in complex with U1 snRNA (right panels). The topographic images of the particles were obtained on the AFM microscope (Nanoscope III) using a contact mode discontinuous with the sample surface. Indicated frame sizes are 1.5×1.5 and 0.6×0.6 µm.

We have assessed the effect of RNA binding on Atcoilin structure and multimerization. Using tryptophan fluorescence we have demonstrated that the addition of U1 snRNA to wt Atcoilin alters its protein structure, such that the emission peak of tryptophan fluorescence is shifted from 347nm to the more hydrophobic 340 nm after complexing RNA (Figure 5A). A similar effect is observed for the NOD, as its peak is shifted to 336 nm, which suggests an increase in the hydrophobicity of the tryptophan local environment (Figure 5A, Table 2). In comparison, the interaction of IDD with RNA does not influence the position of its fluorescence peak (Figure 5A). Fluorescent peak intensity of the full-length Atcoilin and both NOD and IDD domains decreases upon RNA interaction indicating the occurrence of some RNA mediated conformational changes in the protein molecule. Since the changes in peak intensity are maximal in the case of NOD, but much less significant in the case of IDD, it suggests that the modulation of the coilin structure may be predominantly due to the NOD conformational change.

To determine the size of the protein complexes we have used dynamic laser light scattering (DLS), an approach which allows the evaluation of the hydrodynamic radii of particles [36], [37]. The N-terminal part of coilin is required for oligomerization [17], [18]. Indeed, our DLS experiments demonstrate that the NOD determines the oligomerization pattern of coilin (Figure S4). In solution the wt coilin forms 16 nm diameter particles, corresponding to protein oligomers larger than a decamer. Addition of RNA at a protein:RNA molar ratio of 5∶1 leads to the formation of large 50 nm diameter coilin complexes (Figure 5B). With the addition of U1 snRNA to the same ratio (5∶1), the NOD also forms larger particles (250 nm diameter) (Figure 5C). Interestingly, the N-terminal domain forms larger complexes than the wt protein, which may be attributed to the fact that within the full protein the capacity for NOD to oligomerize may be masked by other domains. Thus the change in NOD conformation induced by RNA affects its ability for homologous protein-protein interactions.

In addition, complexes of Atcoilin with U1 snRNA were visualized by AFM (Figure 5D). In the absence of RNA, the coilin sample mainly consists of individual globules with an average height of 1.4±0.4 nm (mean value and mean-square deviation, sample size N = 270) and diameter of 10–25 nm (with tip broadening), which is consistent with the DLS results. The image changes dramatically after the addition of RNA, forming flat lace-like complexes of various sizes with the average height of 0.9±0.2 nm (mean value and mean-square deviation, sample size N = 180) and the mean diameter of 100–200 nm (Figure 5D).

The above data suggests that interaction with RNA not only changes the structure of Atcoilin but also results in its multimerization due to the structural alterations in the NOD.

### Bioinformatic Prediction of NOD Structure

**Figure 6:**
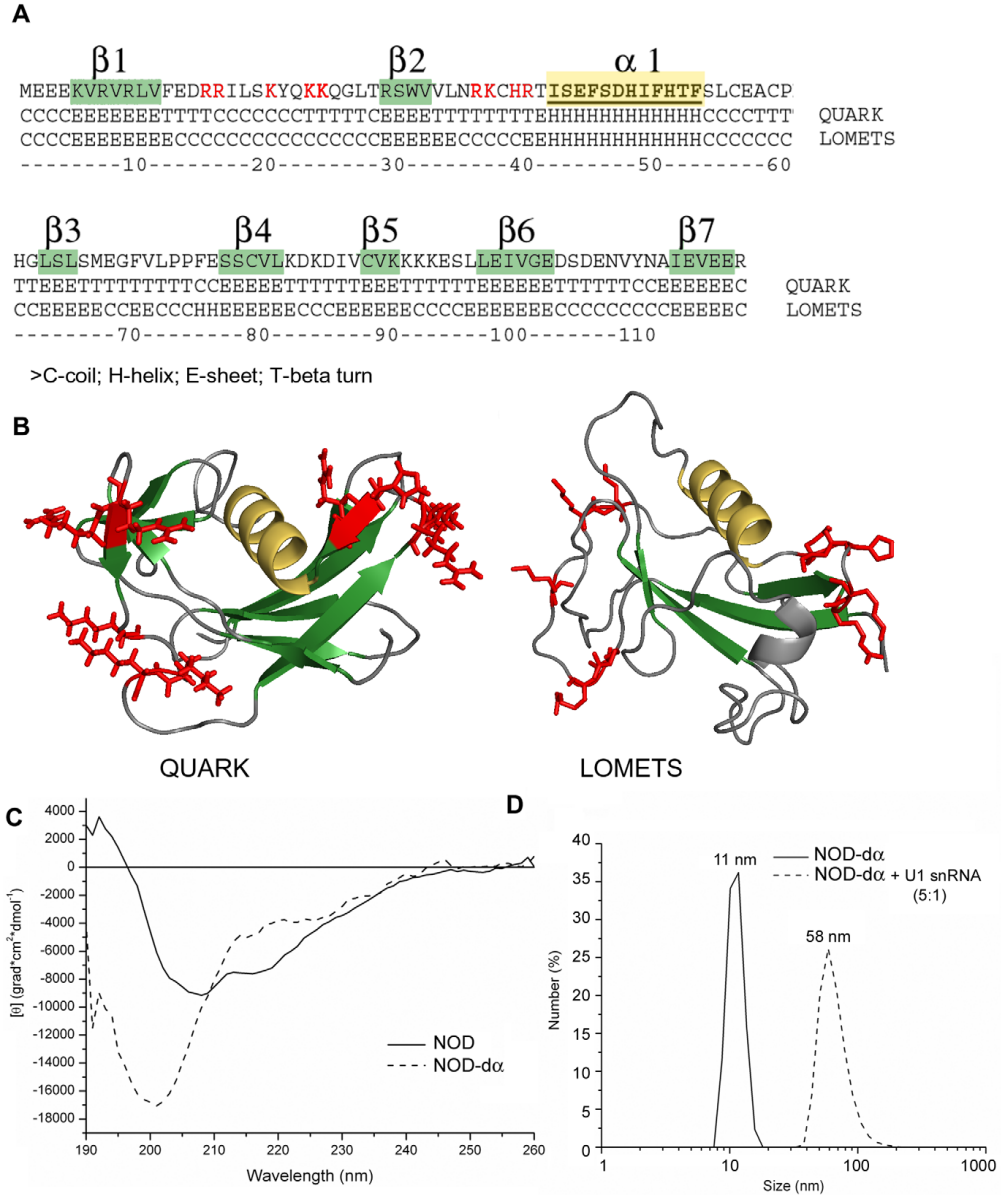
Prediction of NOD tertiary structure. (**A**) NOD protein sequence annotated with the secondary structure elements predicted by the QUARK and LOMETS web-services. (**B**) The three-dimensional model of NOD was constructed using QUARK and LOMETS, with the positive amino acids which replacement with Alanines affect RNA-binding are shown in red letters (A) or in red sticks (B). β-sheets are shown in green, long α-helix is shown in yellow. The picture was prepared using PyMOL (www.pymol.org). (**C**) Circular dichroism far UV-light spectra of NOD and its mutant with the deleted α-helix (NOD-dα). Fluorescence intensity is given in relative units. (**D**) The hydrodynamic radius of the NOD-dα mutant as free protein particles or in complex with U1 snRNA, determined by DLS.

Of the two structured regions within the coilin molecule there is no structural data for NOD. We have used two bioinformatic web-services: QUARK [38] and LOMETS [39] for the prediction of the NOD structure. The results obtained by these two protocols are in good correspondence. In both cases the most probable predicted structure consists of a long α-helix and a subjacent β-layer consisting of several antiparallel β-sheets (Figure 6A, B). Such a topology is referred to as a “ubiquitin-like fold” and is a common structure in many proteins; the U1A protein (U1 snRNP protein) has this structure [40] and interestingly, previous comparisons have been made between it and the N-terminal region of human coilin [3]. It is important to mention that the α-helical content estimated in the models is much lower than that calculated from the CD spectra. This difference may be due to the fact that α-helices in the protein are longer and some residues within them are not taken into account by the program algorithm.

To test the relevance of the model, we have constructed mutant Atcoilin, where one of the basic components of the fold - the long α-helix – was deleted. The secondary structure of the NOD-dα mutant (Figure 3A) was analyzed using CD (Figure 6C). The CD spectrum of this mutant was crucially different from that of the NOD. The N-terminal domain itself shows the spectrum typical for the α/β protein, whence the mutant has the strong negative maximum at 200 nm, which is characteristic for completely unfolded proteins. These data demonstrate that this α-helix might stabilize the NOD tertiary structure. The result supports the model suggested above, since the α-helix was shown to be the crucial element in the formation of the ubiquitin-fold [41], [42].

Addition of U1 snRNA to the NOD with the deleted α-helix (NOD-dα) results in the formation of complexes much smaller than that of the intact NOD combined with U1 snRNA (Figure 6D). The decrease in RNP complex size is not due to perturbation of RNA-binding, since the mutant is more efficient in this activity, than the intact NOD (Figure 3A, 4B). Thus the helix or the tertiary structure of NOD that it stabilizes might affect the multimerization of the coilin.

## Discussion

Bioinformatic analysis has shown that despite the low homology in amino acid sequences, the coilins of different organisms demonstrate a high level of structural and organizational similarity, such that their consistent functionalities may be localized into three main domains. Our data demonstrates that coilin proteins might structurally consist of an N-terminal globular domain (NOD), a central intrinsically disordered domain (IDD) and a C-terminal domain (CTD) containing a presumable Tudor-like structure abutting a disordered C-terminus (Figure S1). The biophysical analysis of recombinant Atcoilin and Atcoilin domain deletion mutants are consistent with these structural predictions; such that the NOD comprises an ordered α/β morphology, the IDD is intrinsically disordered, and the CTD contains a high degree of disorder with a low content of β-structural elements.

In previous works the coilin N-terminus as well as a region in the C-terminal part was defined as being rather conserved across species [6], [7]. We believe that the N-terminal region corresponds to the NOD, which has a globular structure that might be described as an ubiquitin-like fold. Interestingly, earlier work on the expression of coilin phosphomutants reported the formation of an N-terminal degradation product of similar size to the NOD [43], which may further indicate the existence of NOD as a defined domain. The second conserved region, found in the C-terminal part, coincides with the position of the Tudor-like structure (Figure S3B). The Atcoilin CTD contains this structure, and also has a more C-terminal sequence of 100 aa which has a high content of serines (18%) and multiple phosphorylation sites predicted with web service GPS 2.1 [44]. Although in human coilin the sequence C-terminal to the Tudor-like structure is much shorter (20 aa), it also contains multiple phosphorylation sites (at least six), and is required for coilin localization and functioning [22]. Another specific feature of this domain in Atcoilin is the lack of an RGG-box, an important element of animal coilin proteins required for interaction with their main partners – SMN and Sm proteins (for review see [1]).

The exact position of a border between the IDD and CTD is still unclear; on the basis of bioinformatic data we placed it at the 350 aa residue of Atcoilin, but it is quite possible that this border lies around 380–390 aa residues, just before the presumable Tudor-like fold. The central domain is fully intrinsically disordered and highly variable. The increased amount of charged amino acid residues is one of the characteristic features of disordered regions and often the way they are identified [33]. Indeed, the central disordered region contains tracts of alternating charges; for example the strongly negatively charged regions from 94–158 aa (pI 3) and 275–364 aa (pI 4) are interposed by strongly positively charged tracts from 159–274 aa (pI 10), and 365–399 aa (pI 9) (Figure S4). The first two regions (94–158 aa and 159–274 aa, negatively and positively charged regions respectively) were predicted by the MyHits Motif Scan web-service (http://myhits.isb-sib.ch/cgi-bin/motif_scan) to be E-rich and K-rich (Figure 3A). There are two predicted NLSs and a presumable NoLS in the K-rich region. These extensive regions with alternating charges might interact with each other due to the strong electrostatic forces, resulting in masking/embedding of functionally important sites. Phosphorylation may regulate accessibility to these functional sites within the central domain by altering the charges of adjacent tracts. Indeed, such a possibility was checked by introducing mutations into the serine-rich region downstream of the predicted NoLS [18]. The alteration in the charge of the adjacent region resulted in the exposition of the NoLS and redistribution of mutant coilin into the nucleolus [18].

Thus, the structural organization of coilin may be as follows: the protein surface is likely composed of an N-terminal globular domain, the Tudor-like structure and the adjacent unfolded regions. The major part of the disordered IDD is involved in intramolecular interactions, likely forming a kind of a protein “stick” (Figure S5).

In order to find out whether the features are specific for Atcoilin or are also present in coilin proteins of other plants, we aligned a number of plant proteins showing more than 39% homology with Atcoilin in the Basic BLAST search (http://blast.ncbi.nlm.nih.gov/Blast.cgi) (Figure S6). These proteins have not yet been identified as coilin, so we refer to them as “coilin-like proteins”. The alignment analysis revealed high homology in the N-terminal region (about 1–90 aa), including the RNA-binding site (R15, R16, K20, K23, K24) which is absent from the animal coilin protein. Although the analogous IDD sequences found in the coilin-like proteins have high sequence and size variation, they share a similar alternating charge pattern at corresponding regions to those identified in the internal domain of Atcoilin (Figure S6). The C-terminal parts of these proteins demonstrate a great deal of overall homology and interestingly they share a completely identical octamer KKKGQKWG (368–375 aa in Atcoilin); it can be suggested that this motif may be a plant analog of an RGG-box. The C-terminal regions also contained elements of pronounced similarity that were homologous to a Tudor-like region typically found in human coilin (Figure S3B, S6). Moreover, all plant coilin-like proteins have an extensive disordered (about 100 aa) serine-rich sequence on the most C-terminal part of the molecule. Taken together this indicates that the C-terminal domain of plant coilins might be composed of two subdomains. The analysis of the amino acid sequences of these proteins with Foldindex and Disopred, revealed the same pattern of folded/unfolded regions to that of Atcoilin (data not shown). Thus, all the specific features of Atcoilin are likely to be common properties of plant coilin-like proteins.

Atcoilin demonstrates effective and non-specific RNA-binding activity. This activity is provided by three sites of positively charged amino acids - two in the NOD and one in the central IDD (the predicted NLS2). All these sites are localized in disordered regions on the surface of the molecule. Both NOD RNA-binding sites, according to the structural models, are located in the loops (Figure 6B, LOMETS model) or/and in the β-sheets not incorporated into the β-layer (Figure 6B, QUARK model). The topology of the NOD RNA-binding sites are quite similar in both models; they are on the opposite ends of the curved β-layer separated by the α-helix.

The RNA-binding and multimerization of coilin seem to be connected. The RNA-binding pattern of various Atcoilin mutants points to the fact that the oligomerization region lies within NOD (14–78 aa), a region which is key for RNA binding. It has been demonstrated that interaction of NOD and full-length coilin with RNA leads to multimerization/aggregation of these proteins, a process induced by the structural remodeling of NOD. The interaction of NOD with RNA may be one of the factors that induce coilin to switch from a soluble nucleoplasmic state into an aggregated complex that likely accumulates to form Cajal bodies. It is suggested that CBs facilitate snRNP biogenesis by providing a locally high concentration of the active molecules [45]. It is known that proper formation of functional CBs depends on multiple factors [1], such as the interaction and immobilization of coilin on DNA [46]. It is therefore probable that the Atcoilin-RNA interaction might be an additional key factor, which facilitates proper assembly of a scaffold required for correct CB formation.

## Supporting Information

Figure S1 Comparison of the FoldIndex predicted folded/unfolded regions of coilin proteins from different species.
(TIF)

Figure S2 SDS-PAGE of Atcoilin and its isolated domains (NOD, IDD and CTD) expressed and purified from *E. coli* (Coomassie blue staining).
(TIF)

Figure S3 Sequence alignment of the most conserved coilin protein sequences from *Arabidopsis thaliana* (NM_101173.4; GI:42562030**)** and *Homo sapiens* (NP_004636.1; GI:4758024). **(A)** Alignment of predicted NODs from these two proteins, which have a similarity score of 30%. The positive amino acids whose replacement with Alanines affect RNA-binding, are shown in bold. **(B)** Alignment between the Tudor-like fold of human coilin and the homologous sequence from *Arabidopsis thaliana* coilin. The similarity score between the compared sequences is 45%. The alignment was performed by CLUSTAW2 multiple sequence alignment (http://www.ebi.ac.uk/Tools/msa/clustalw2/). Asterisks mark the identical residues in all sequences, colons denote conserved substitutions, and dots highlight the semi-conserved substitutions.
(TIF)

Figure S4 Influence of salt concentration on the pattern of the oligomerization of coilin and its isolated domains. DLS measurement of the hydrodynamic radius was used to determine the level aggregation of Atcoilin (A), NOD (B), IDD (C) and CTD (D) after exposure to different NaCl concentrations. Atcoilin and NOD have similar levels of resistance to salt induced aggregation, whereas IDD and CTD were much more susceptible to aggregation upon salt exposure (precipitation occurred at 50 mM NaCl).
(TIF)

Figure S5 The putative structural organisation of the Atcoilin molecule. Domains and functionally important regions are indicated. NLS1, NLS2 and 7K (cryptic NoLS) are indicated as black boxes. The alternate charged regions within the central part of the protein are indicated by “+” or “−”, according to their charge. Folded regions are shown as spheres.
(PDF)

Figure S6 Sequence alignment of *Arabidopsis thaliana* coilin and several coilin-like proteins from various plant species. The protein sequences were obtained by using *Arabidopsis* coilin as a query in a Basic protein BLAST search (http://blast.ncbi.nlm.nih.gov/Blast.cgi). Plant proteins with the highest homology ratios (above 39) were selected for the alignment. We refer to them as coilin-like proteins: Brassica (*Brassica rapa*; ABQ50545.1), Ricinus (*Ricinus communis*; XP_002530050.1), Populus (*Populus trichocarpa*; XP_002315658.1), Vitis (*Vitis vinifera*, CBI16805.3), Medicago (*Medicago truncatula*, XP_003601896.1), Brachypodium (*Brachypodium distachyon*, XP_003576896.1). The alignment was performed using the CLUSTAW2 multiple sequence alignment tool. Asterisks mark identical residues in all sequences, colons indicate conserved substitutions, dots denote the semi-conserved substitutions. Extensive charged regions are marked in blue (negatively charged), red (positively charged) and yellow (neutral). The positive amino acids whose replacement with Alanines affect RNA binding of different domains in the *Arabidopsis thaliana* coilin (and the homologous aa residues in other proteins) are shown as white letters on black and the aa residues homologous to them are highlighted in gray. Conserved octamer KKKGQKWG is shown in a box.
(TIF)
