# Supplementary figures and images for: Plant Coilin: Structural Characteristics and RNA-Binding Properties
Source: PLoS One. 2013 Jan 8;8(1):e53571. doi: 10.1371/journal.pone.0053571 (PMC3539977; doi:10.1371/journal.pone.0053571)

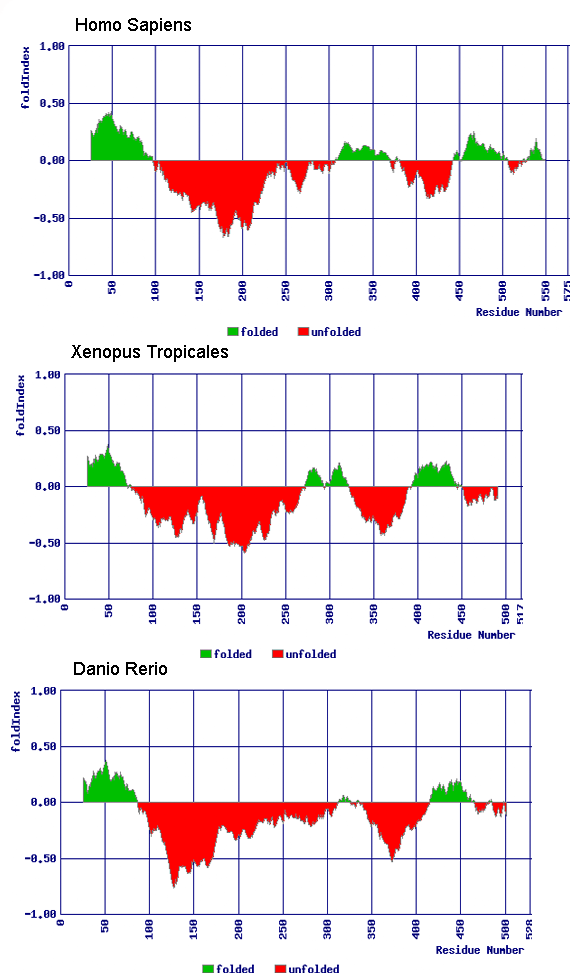

Supplement: Figure S1 — Comparison of the FoldIndex predicted folded/unfolded regions of coilin proteins from different species. (TIF) [file pone.0053571.s001.tif]

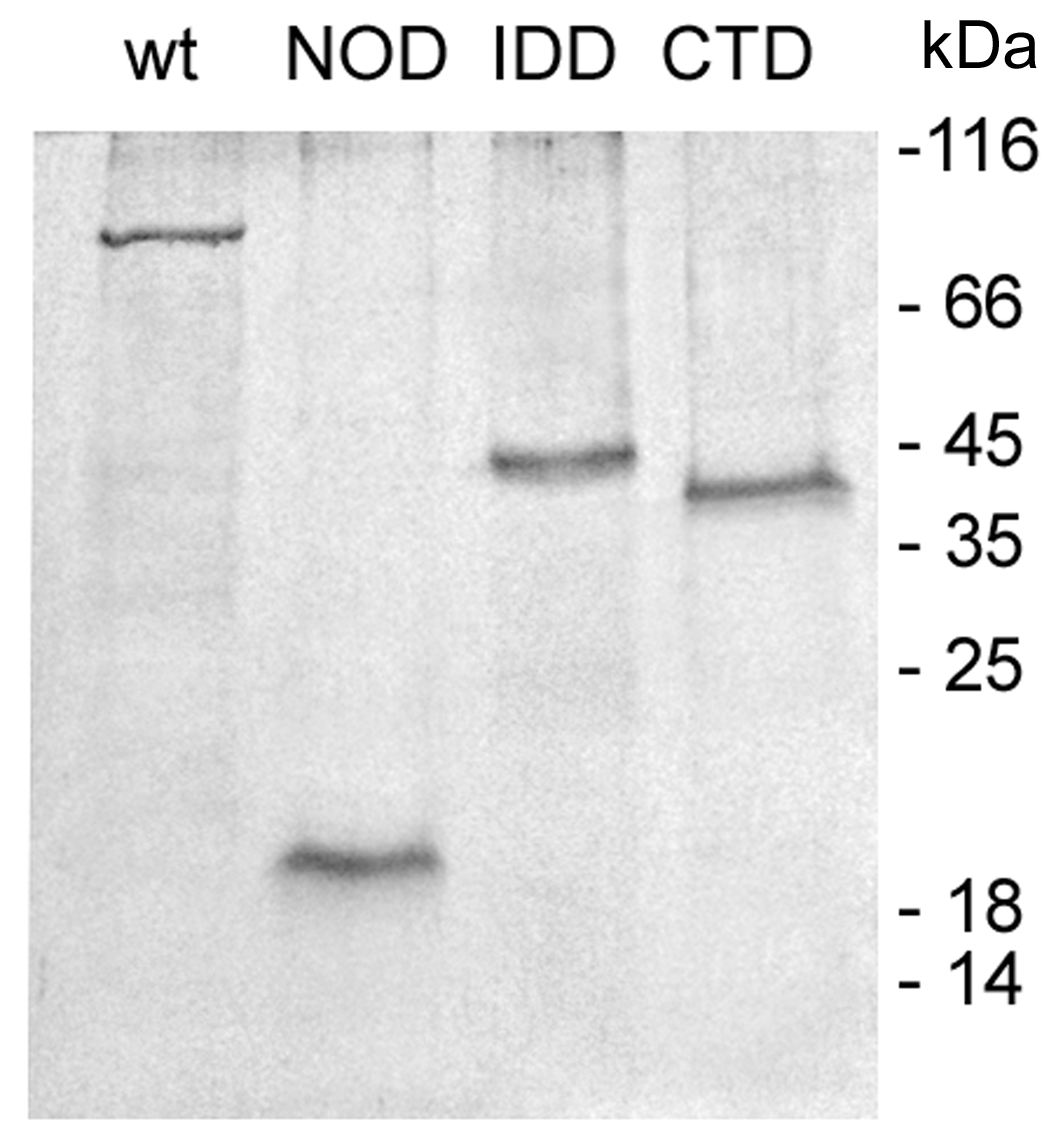

Supplement: Figure S2 — SDS-PAGE of Atcoilin and its isolated domains (NOD, IDD and CTD) expressed and purified from E. coli (Coomassie blue staining). (TIF) [file pone.0053571.s002.tif]

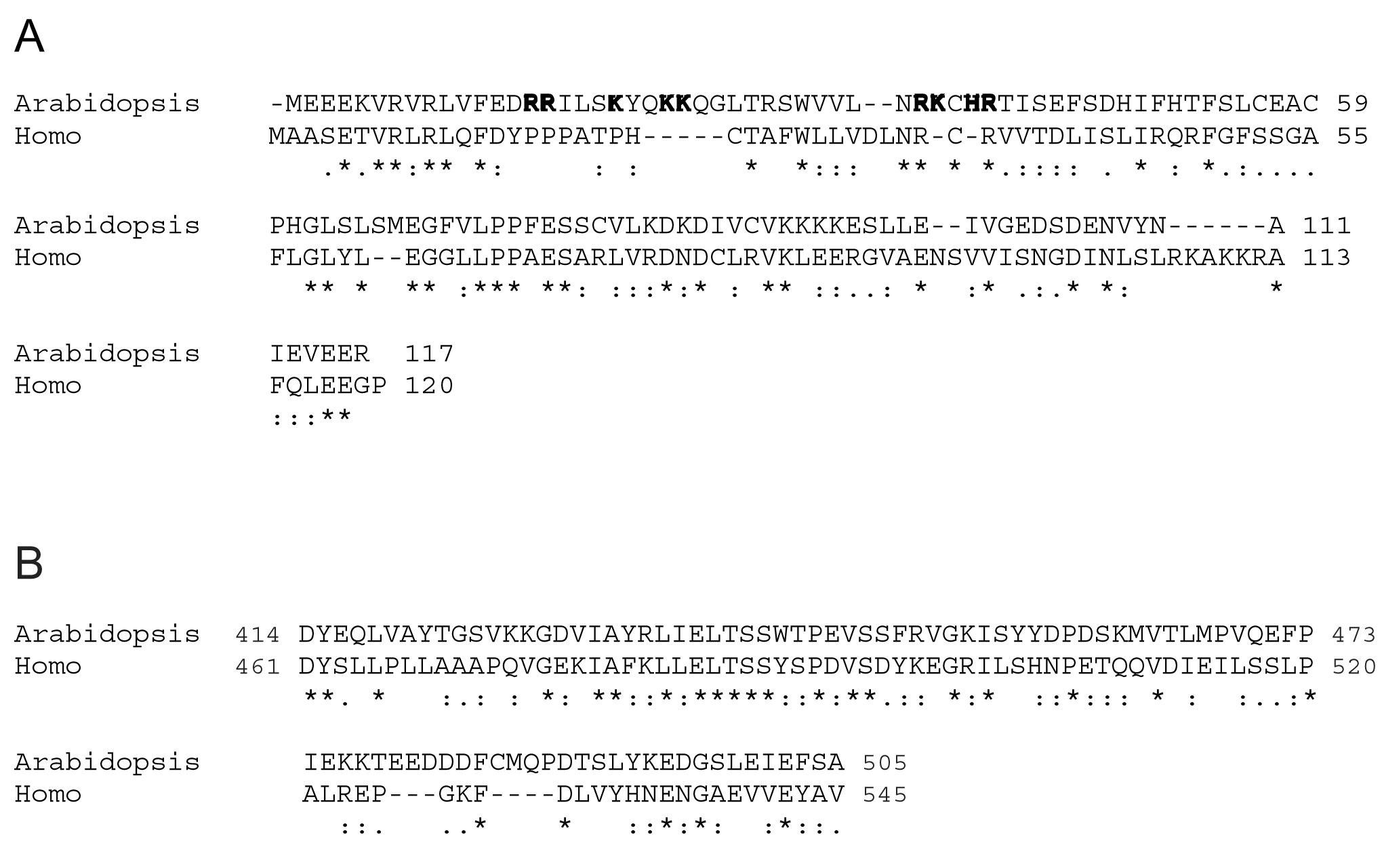

Supplement: Figure S3 — Sequence alignment of the most conserved coilin protein sequences from Arabidopsis thaliana (NM_101173.4; GI:42562030) and Homo sapiens (NP_004636.1; GI:4758024). (A) Alignment of predicted NODs from these two proteins, which have a similarity score of 30%. The positive amino acids whose replacement with Alanines affect RNA-binding, are shown in bold. (B) Alignment between the Tudor-like fold of human coilin and the homologous sequence from Arabidopsis thaliana coilin. The similarity score between the compared sequences is 45%. The alignment was performed by CLUSTAW2 multiple sequence alignment (http://www.ebi.ac.uk/Tools/msa/clustalw2/). Asterisks mark the identical residues in all sequences, colons denote conserved substitutions, and dots highlight the semi-conserved substitutions. (TIF) [file pone.0053571.s003.tif]

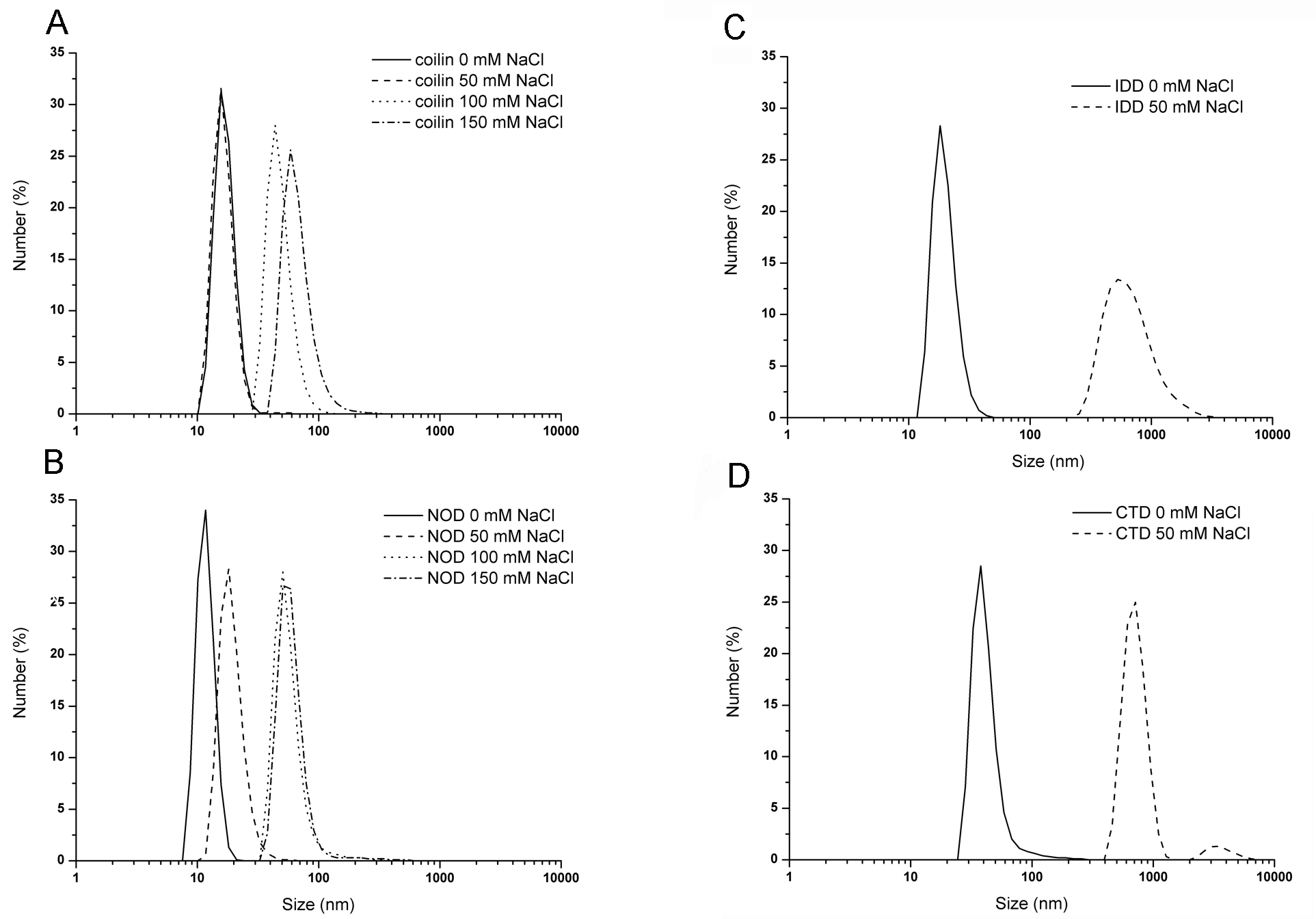

Supplement: Figure S4 — Influence of salt concentration on the pattern of the oligomerization of coilin and its isolated domains. DLS measurement of the hydrodynamic radius was used to determine the level aggregation of Atcoilin (A), NOD (B), IDD (C) and CTD (D) after exposure to different NaCl concentrations. Atcoilin and NOD have similar levels of resistance to salt induced aggregation, whereas IDD and CTD were much more susceptible to aggregation upon salt exposure (precipitation occurred at 50 mM NaCl). (TIF) [file pone.0053571.s004.tif]

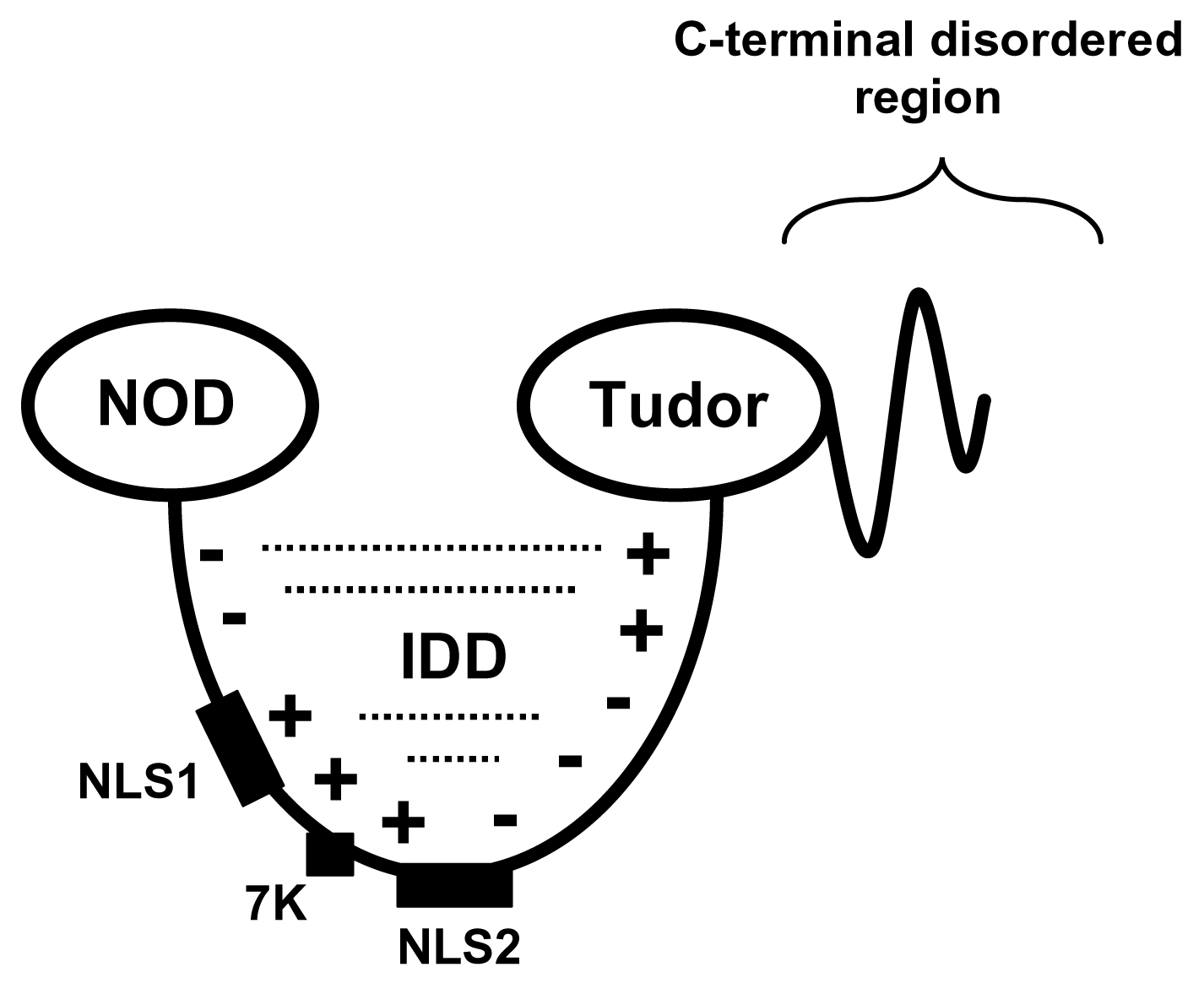

Supplement: Figure S6 — Sequence alignment of Arabidopsis thaliana coilin and several coilin-like proteins from various plant species. The protein sequences were obtained by using Arabidopsis coilin as a query in a Basic protein BLAST search (http://blast.ncbi.nlm.nih.gov/Blast.cgi). Plant proteins with the highest homology ratios (above 39) were selected for the alignment. We refer to them as coilin-like proteins: Brassica (Brassica rapa; ABQ50545.1), Ricinus (Ricinus communis; XP_002530050.1), Populus (Populus trichocarpa; XP_002315658.1), Vitis (Vitis vinifera, CBI16805.3), Medicago (Medicago truncatula, XP_003601896.1), Brachypodium (Brachypodium distachyon, XP_003576896.1). The alignment was performed using the CLUSTAW2 multiple sequence alignment tool. Asterisks mark identical residues in all sequences, colons indicate conserved substitutions, dots denote the semi-conserved substitutions. Extensive charged regions are marked in blue (negatively charged), red (positively charged) and yellow (neutral). The positive amino acids whose replacement with Alanines affect RNA binding of different domains in the Arabidopsis thaliana coilin (and the homologous aa residues in other proteins) are shown as white letters on black and the aa residues homologous to them are highlighted in gray. Conserved octamer KKKGQKWG is shown in a box. (TIF) [file pone.0053571.s006.tif]
