# Supplementary material for: Plant Coilin: Structural Characteristics and RNA-Binding Properties
Source: PLoS One. 2013 Jan 8;8(1):e53571. doi: 10.1371/journal.pone.0053571 (PMC3539977; doi:10.1371/journal.pone.0053571)
Supplement: Figure S5 — The putative structural organisation of the Atcoilin molecule. Domains and functionally important regions are indicated. NLS1, NLS2 and 7K (cryptic NoLS) are indicated as black boxes. The alternate charged regions within the central part of the protein are indicated by “+” or “−”, according to their charge. Folded regions are shown as spheres. (PDF) [file pone.0053571.s005.pdf]

```
Arabidopsis       ------------MEEEKVRVRLVFEDRRILSKYQKKQGLTRSWVVLNRKCHRTISEFSDH 48
Brassica          ------------MAEETVRIRLVFENQRILSKHQKKQGLKRSWAVLNPKYHRTVSEFSNH 48
Ricinus           --------------METVRLRLVFD--QILSKVQNTQGLKRCWILLKPQ-HQTISDLSSY 43
Populus           --------------METVRLRLVLD--NALT--TTTEGLRKCWILLKPQ-HKTISDLSSH 41
Vitis             ------------MAMETVRVRVVLEDPDLLNKTQNSEGLRRSWLLLKPQ-HKTISDLSSY 47
Medicago          MVTESDKTATLTMMSNKVRLRLLFDDRNMLSKSKKKEGLKCCWFLLKPH-LTTISDLASH 59
Brachypodium      --------MATPPAPSPVRLRLLFDNRRLLRRAQRDDGLRRCWLLLRPE-LETVADLSAH 51
                                 . **:*::::    *      :**  .* :*. .   *::::: :

Arabidopsis       IFHTFSLCEACPHGLSLSMEGFVLPPFESSCVLKDKDIVCVKKKKESLLEIVGEDSDEN- 107
Brassica          ILYTFSLFEACPHGLSLYMDGFVLPPFESSCVLKDKDIVCVKRKKEPLLEIVGEDSEEN- 107
Ricinus           LLNVFNLQNHCPHGLLLLMEGFALPGFECTSILKDKDIIRVEKNGGVSSEIVMVGDDVNN 103
Populus           ILQVFDLHNACPSGLFLSMEGFVLPPFESTCILNDKDIVRVIKKDGTATEIIKIDDGLND 101
Vitis             LLRIFNLHDFCPNGLLLSMDGFVLPSFESTCILKDKEIISVKRKGGAVIDLLEVGDETNC 107
Medicago          ILAVFRLHRNCPDGITLSMDGFVLPSFESTCILKDKDIVCVKKKGSMLTGSKP------- 112
Brachypodium      VAARFRLRRSCPHGIVFSMDGFALPPFESTCIFRDKDIIRVKQK--SCKNIVHHNAVHCI 109
                  :   * *   ** *: : *:**.** **.:.::.**:*: * ::

Arabidopsis       VYNAIEVEER-PQIRPGEMLLANEEFQKETGGYESESEEDE--LEEE-----AEEFVPEK 159
Brassica          VCAAIEIEERRARICPDAVLIGNEE----TGGYESESEEE-------------EEIVPEK 150
Ricinus           ALEVVEIVETQPLVTTGMNLLANEEFEKESGGYQSDEEEDVPKQEEEA-VHVVENASEVK 162
Populus           SLNVVEIID-KPPVTTGTNLLANEEFEKESDSYESEQEVDVAAEVED-----VENSPEVK 155
Vitis             SEDEAIVEN--QHIHRGVKLLANEEFDKETGGYESESEEDEPDQPEE--TVQVETASAGN 163
Medicago          --AMLPSETHENQLIELPKLLAIEGFQEKREEYETISEDDDDDNDQSEDVVNVESKLDGN 170
Brachypodium      QEPEIVEKRPLPTDEKILAIEYQMDCGKHQEEVHCEYQSEE------------NATSNQD 157
                                     :             .   : :             :     .

Arabidopsis       KASKKRKTSSKNQSTKRKKCKLDTTEESPDERENTAVVSNVVKKKKKKKSLDVQSANN-- 217
Brassica          KTSKKRP-------------RFKKRAMSPDE----------------------------- 168
Ricinus           TISKKRKAMKDLKSPKRKKTKSARAEKHAAVLEDMGNNVTGEQNGTSCTPKFDERS---- 218
Populus           AVSKKRKASKDAHSPKRKKTKSASAKKCVEVSENVVTDVSAEQNGTLGIVDVDERSGKSR 215
Vitis             AGSKKRKASRKLKSPKRKKNKYTRLEKCPVVLEDVENGVCEEQTKSCDDCTALPKKGSLK 223
Medicago          ATSKKRKASKKLKSPSKKKIKMSTTENLADIPEAHEEENGSVKDCTHRKASLAKKDIG-- 228
Brachypodium      TSSKRKQRDGDAGRPESLKRKKLKVTTPDNVCQDRVHCSSNNSKPSTIDAEAKKGAS--- 214
                    **::              :

Arabidopsis       --------------------------------------------DEQNNDSTKPMTKSKR 233
Brassica          ------------------------------------------------------------
Ricinus           -------------------------------------------------ESSKALSKAKR 229
Populus           KAMRDTKKRKKTKSASAEKCMEVSENVVTSVCAGQNGTLSFFKADERSSKSTEATINAQK 275
Vitis             KHKSSNVNGKPDKARTLN-------------------------IDERSNDVDESSPNAKR 258
Medicago          ---------------------------------------------------KSSTLSCQK 237
Brachypodium      ------------------------------------------------------------

Arabidopsis       SSQ-QEESKEHNDLCQLSAETK-------------------------------------- 254
Brassica          -------------LCIMSAETK-------------------------------------- 177
Ricinus           ISQPQENGDASVDASPSTSGTK-------------------------------------- 251
Populus           SCQPEQNGNGSVDASHILSGSKKVLIYHFTEHMANICVFNSEMCCSLSLRNWVKLSMMLG 335
Vitis             CGELQENGSQGVEVANPPDGTQ-------------------------------------- 280
Medicago          NDKSGSTSEETRSLQPQDEGEPK------------------------------------- 260
Brachypodium      ----QAEGIVRLDEKQKTDSVCT------------------------------------- 233

Arabidopsis       ---------------------------------KTPSRSARRKKAKRQWLREKTKLEKEE 281
Brassica          ---------------------------------KLQQN---------------------- 182
Ricinus           ---------------------------------KLPSRSARRKQAKRRWLREKLRAERKE 278
Populus           VLQVWPVDQGAQARGYWPPYASNDSNTCISLSCQCPSRSARRKKAKRHWLKEQLKAEKKA 395
Vitis             ---------------------------------KYPSRSARRKKAKRKWLRELAKVEKKE 307
Medicago          ---------------------------------KLPSRSARRKKAKRKWLRELKLKEKEK 287
Brachypodium      ---------------------------------SCQSRSARRKKLKRQLKKKAKEELKEN 260
                                                   .  ..
```

```
Arabidopsis       LL------QTQLVVAPSQKPVITIDHQATKEK----HCETLENQQAEEVSDGFGDEVVPV 331
Brassica          --------QMQMVVAPSKKPAITIDHQETEEN----RSEALEKQQPDEQGDGVRDEVV-- 228
Ricinus           QS--------SQTLAEKGNNGVFEELPESGG-----EEPQEDDQQPDKDSDLEDD-IVPI 324
Populus           QNKRELLLNINQQSSEKDNQNVSGESPEPGSQKTSEEKLREDNQLGERDSDVEGD-VVPV 454
Vitis             MH--------QRQSPEKEVQ-----------------KNSLEHQQPDQNSDTDDAVIVPI 342
Medicago          EKE-----KENENEKDKLHPSQVLEKDDQQIPIKDNNGKVSDVRQQSNEESEADDDMVPV 342
Brachypodium      GHCQ-----EPPIAADCPPSSNQDDLCPSSNQKDPHLPFSSHEAEAEEEESETADDIVPV 315
                                                                           .    ..  .     :*
                                                             “RGG”
Arabidopsis       EVRPGHIRFKPLAG------TDEASLDSEPLVENVLWNGNMTKKKGQKWGTEK-SGFSKR 384
Brassica          ----------------------------VPVEESFLWNGNMTKKKGQKWGTEK-TGFSKR 259
Ricinus           VIRPGHIRFEPLSK----AGANQAEQQTHIPVEIFHWNGITSKKKGQKWGKEKATSYKRN 380
Populus           VIRPGHIRFEPLKKGLMLGDSDHAVPQNHVSIERFQWNGITSKKKGQKWGKEKVVSCKRN 514
Vitis             VIRPGHIRFEPLGK-------DQTIQQNPVSVETFQWNGTTSKKKGQKWGKEK-MSCRRN 394
Medicago          EIRPGHIRFQPRGK-------DLAAPENQLPVDTFQWNGTTSKKKGQKWGKERTSSHKQD 395
Brachypodium      VVRPGHIRFEPAGIS-----RERSTSSAKEIQGTFTWSGTMSKKKGQKWGMNNSNKKSAD 370
                                                    . *.*  :******** :.

Arabidopsis       YAQDFNED-ATTQPA--------------------------------------------- 398
Brassica          YAWDLNDTYHQTQPA--------------------------------------------- 274
Ricinus           DHRNVS-QECSEVRK--------------------------------------------- 394
Populus           DYNNFKKESYSSLTI--------------------------------------------- 529
Vitis             DYKDFNQQHSETFAV--------------------------------------------- 409
Medicago          DYEPSSQDGHAAQWRGTVSKKDPKWGKERTSSHKQNDYEQSSQDGRTVQWSGTASKKKDQ 455
Brachypodium      IGHVGKVAGTNTEVNHLVLDSKNEENGFCG------------------------------ 400
                       .

Arabidopsis       ------------------------------------------------------------
Brassica          ------------------------------------------------------------
Ricinus           ------------------------------------------------------------
Populus           ------------------------------------------------------------
Vitis             ------------------------------------------------------------
Medicago          IWGKGWTSSHKQDDHEQSSQDRPTVQRNGTISKDQKWGKEKTSAHKHDHEQSSQDRPTVQ 515
Brachypodium      ----------------------------------------------------------VS 402

Arabidopsis       ------------EAETLAN------------------------CPIDYEQLVAYTGSVKK 422
Brassica          ------------EAETLAN------------------------GQIDYEQLVAYTGSVKK 298
Ricinus           ------------DGGRPVY------------------------DRVDFEKLEFYTTLPKE 418
Populus           ------------EEQTPVY------------------------DCTNFEEFPLYASLPKE 553
Vitis             ------------EEGTPPK------------------------DPMDFDKLPSLTSSPKE 433
Medicago          WNGTISKDQKWGKEKTSAHKQDDCEQSSQDRPTDQNAGKKRTFDAVDFEKLTPYTDLPKE 575
Brachypodium      NQIVESSHDVLLREKTHAEEG------------------KSISESMDFDSLYPLTRLPKE 444
                                                                ::::.:   :   *:

Arabidopsis       GDVIAYRLIELTSSWTPEVSSFR--------------------------VGKISYYDPDS 456
Brassica          GDIIAYRLIELTSSWTPEVSSFRVKFLTLLFEPVDQSKYLSRLRYVHLLVGKISYYDPVS 358
Ricinus           GDVIAYRLIELSPSWTPEISSYR--------------------------VGKISRYDMQS 452
Populus           GDVIAYRLVELSSSWTPELSSYR--------------------------VGKVSKYDLES 587
Vitis             GDMIAYRLIELSSTWTPELSTFR--------------------------VGKISSYDPES 467
Medicago          GNVIAYRLIELSESWTPELSSFR--------------------------VGKTTQYDSKS 609
Brachypodium      GDLIVYRLVELSSSWCPEISSYR--------------------------VGKVLIYDLIS 478
                  *::*.***:**: :* **:*::*                          ***   **  *

Arabidopsis       KMVTLMPVQEFPIEK-KTEEDDDFCMQ---PDTSLYKEDG----SLEIEFSALLDVRSVK 508
Brassica          KMVTLMPVQGYPIEK-KIEEDDDSSMQ---ADTSLYNEDG----SLEVEFSSLLDVRSVK 410
Ricinus           NRVRLVPVPGYPVTH-KETGDDASAAP---SETIPYAKDG----SLWIEFASLIEVRLVT 504
Populus           NIVMLAQVPEYPVIP-EKIDDEASDAL---PETSPYQDDGSLEVGVSIKFSALFEVRLVH 643
Vitis             NKLILISVPESPIVA-ETRIDEDASALEPDPDTSLYREDG----SLEIDFSSLIDVRIIK 522
Medicago          NRIWLQPVSEFPFDFWKKIDDMDEDGSPSQSDPSPYQEDG----SLEIDYASLADVRIIK 665
Brachypodium      MRIILLPVPEYPIIKEETAGEDESDMP---VDMSPYSEDG----SLEIEYSSLLDVRLLK 531
                    : *  *   *.   :   :          :   * .**    .: :.:::* :** :

Arabidopsis       TSSSDSAEVAKSA---L-PEPDQSAKKPKLSAN--------------------------- 537
```

```
Brassica          TSSSDVVEVAT--------KPDQAATNLKLSTNS------------------------- 436
Ricinus           RGNSNSVKSVAGEIDKV-PVRDQDNRTGCRSNN-------------------------- 536
Populus           HGNIKSAKSVTGGSNEV-HVRDQDSGTGFKLNN-------------------------- 675
Vitis             SGNSHLEKAVTAR-VEA-PVDTQDAVSGVKPNNKNSGMSTSLPGGELNITQVSVAGVEHN 580
Medicago          HGHSDLATVVAHSDAFVTPTKATNNSTDEKPADN------------------ETAAGSSK 707
Brachypodium      GSESVSTAVSTPIRETGKKGESLVKQPVTLDKNK--------------------GVIHSQ 571
                   .                              :

Arabidopsis       --KELQTPAKENGEVSPWEELSEALSAKKAALSQANNGWNKKG-SSSGGSWSYKALRGSA 594
Brassica          --NGLQTTVKENGKGNPWEELSEAVSAKKAKLSEANNGWNKKGKSSSGGSWR----RGGG 490
Ricinus           -GNESHVSAQENGKGNAWEEISEALNAKKAELAQEDN-WNKPG-SSGRRPWSYKALRGSA 593
Populus           -NHEAGTSAQENGKHNPWEETNQALTAQKAWLSQEDS-CKKPE-SSGRSPWSYKALRGSA 732
Vitis             INREMTAPPPENGKVNAWDEIDKVLSAKKAQLSQEDG-SSKKE-SPGRSPWSYKALRGSA 638
Medicago          PQIEGHVTAKENGEVNVWDEINEALKAKKTRLSQEDR-WKKEGDSSENRSWSQRALRCSA 766
Brachypodium      TEPLVPNNTKDPEANKIWDESIESLSDKPDEVQENGWGTWKPN--SSTSAWSYRAQRSTA 629
                            :    . *:*  : :. :   : : .    *    .   .*     *  .

Arabidopsis       MGPVMNYLRSQKEI------------- 608
Brassica          IGPLMNYLRSQKEI------------- 504
Ricinus           LGPTMALLRAQNEL------------- 607
Populus           LGPTVALLRAQNEL------------- 746
Vitis             LGPTMSFLRAQNNF------------- 652
Medicago          LGPTMALLRSQNGFKK----------- 782
Brachypodium      LGPTLALLRGKNGKGGKAKPPNRKYGK 656
                  :** :  **.::
```
